# Structural color printing via polymer-assisted photochemical deposition

**DOI:** 10.1038/s41377-022-00776-x

**Published:** 2022-04-06

**Authors:** Shinhyuk Choi, Zhi Zhao, Jiawei Zuo, Hossain Mansur Resalat Faruque, Yu Yao, Chao Wang

**Affiliations:** 1 School of Electrical, Computer and Energy Engineering, Arizona State University, Tempe, AZ 85287 USA; 2 Biodesign Center for Molecular Design and Biomimetics, Arizona State University, Tempe, AZ 85287 USA; 3 Present Address: College of Materials Science and Engineering, Key Laboratory of Advanced Functional Materials, Education Ministry of China, Beijing University of Technology, Beijing, 100124 China

**Keywords:** Nanophotonics and plasmonics, Displays

## Abstract

Structural color printings have broad applications due to their advantages of long-term sustainability, eco-friendly manufacturing, and ultra-high resolution. However, most of them require costly and time-consuming fabrication processes from nanolithography to vacuum deposition and etching. Here, we demonstrate a new color printing technology based on polymer-assisted photochemical metal deposition (PPD), a room temperature, ambient, and additive manufacturing process without requiring heating, vacuum deposition or etching. The PPD-printed silver films comprise densely aggregated silver nanoparticles filled with a small amount (estimated <20% volume) of polymers, producing a smooth surface (roughness 2.5 nm) even better than vacuum-deposited silver films (roughness 2.8 nm) at ~4 nm thickness. Further, the printed composite films have a much larger effective refractive index *n* (~1.90) and a smaller extinction coefficient *k* (~0.92) than PVD ones in the visible wavelength range (400 to 800 nm), therefore modulating the surface reflection and the phase accumulation. The capability of PPD in printing both ultra-thin (~5 nm) composite films and highly reflective thicker film greatly benefit the design and construction of multilayered Fabry–Perot (FP) cavity structures to exhibit vivid and saturated colors. We demonstrated programmed printing of complex pictures of different color schemes at a high spatial resolution of ~6.5 μm by three-dimensionally modulating the top composite film geometries and dielectric spacer thicknesses (75 to 200 nm). Finally, PPD-based color picture printing is demonstrated on a wide range of substrates, including glass, PDMS, and plastic, proving its broad potential in future applications from security labeling to color displays.

## Abstract (web summary)

Micro-scaled structural color printings are demonstrated by light-programmed, solution-phase, additive manufacturing technique at ambient conditions without complex lithography or etching steps.

## Graphical abstract

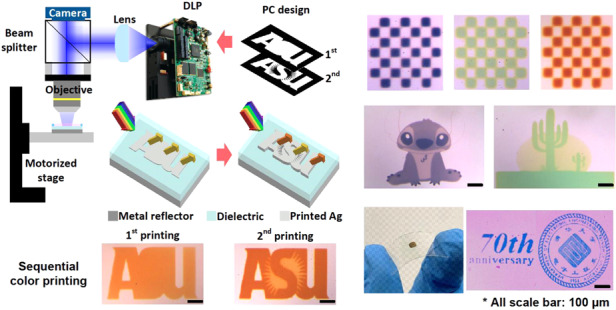

## Introduction

Colorful pigments from pictures serve to selectively absorb light within a spectral range, thus modulating the light reflection and color display. Conventional pigments, however, are usually toxic and relatively large. They also tend to degrade over time and lose their brightness and resolution. As an alternative, structural color makes use of micro- or nano-structured materials, such as plasmonic nanoantennas (i.e., disks, holes, and rods)^1–3^, metasurfaces^4–6^, photonic crystals^7,8^, and thin-film interferometer^9–11^, to modulate the light absorption, scattering, and interference and accordingly display color. For example, plasmonic nanoantennas make use of localized surface plasmon resonance (LSPR) to engineer light absorption and scattering at a sub-wavelength scale, capable of achieving ultra-high printing resolution. Dielectric (e.g., silicon^12,13^, titanium oxide^14^, etc.) nanoantennas exploit geometry-dependent electric dipole and magnetic dipole resonances that generate strong Mie resonance in the visible wavelength range while exhibiting much lower optical losses compared to plasmonic nanostructures. Fabry–Perot (FP) cavity^15^, typically employing a sandwich thin-film structure compromising a dielectric spacer between two reflectors, produces interferences between the top and bottom reflectors, leading to a strong resonance in reflection magnified as a color change^16–21^. These metallic and dielectric structures are more durable than organic pigments^22,23^, and can be scalably produced using eco-friendly manufacturing technologies^24,25^. As a result, structural color has emerged as a promising alternative for various color-display applications including filters^26–28^, holograms^29,30^, colorimetric sensors^31,32^, anti-counterfeiting^33,34^, and decoration^35,36^.

To engineer optical resonance at particular wavelengths, structural color technologies based on plasmonic and dielectric nanoantennas require a precise definition of structural dimensions at the nanometer scale. The nanofabrication processes, such as electron beam lithography (EBL), focused ion beam (FIB), physical vapor deposition (PVD), and reactive ion etching (RIE), however, can seriously increase the cost and limit the applications. In comparison, conventional FP cavity structures use much simpler fabrication processes, mainly involving vacuum deposition of metal-dielectric-metal film stacks. Additionally, the optical resonance and color display can be conveniently tuned by modifying the thickness and/or refractive indexes of dielectric and metallic layers. However, there are still several remaining challenges for low-cost, high-resolution, and at-demand color printing of the FP cavity structures. First, conventional metal-based high-quality FP cavity presents a very sharp absorption with a broadband non-resonant reflection as background, which leads to low brightness and poor color purity in reflection^37–39^. The color saturation performance could be greatly improved by intentionally lowering the qualify (Q) factor of the cavity resonance, e.g. by using an absorbent material such as germanium (Ge) and nickel (Ni) as the top reflector^40,41^. Further, the fabrication of such a thin-film stack comprising different materials requires multiple vacuum-based deposition processes in specialized facilities. This typically results in a long processing time and increased cost. Additionally, the metal deposition process generally is associated with high temperature but is not always compatible with organic and soft materials, thus constraining the color printing applications on flexible substrates. Lastly, high-resolution printing requires micro-lithography in a well-controlled cleanroom environment, which is not readily accessible and potentially costly.

In this work, we will demonstrate the use of accessible, inexpensive, additive manufacturing (AM) technology to create colored pictures without photolithography, vacuum deposition, or etching processes. Here, we utilize our recently developed polymer-assisted photochemical deposition (PPD) process^42^ to print the metallic reflectors in a FP cavity. The PPD technology is a room temperature, non-toxic, solution-based additive manufacturing process, and is used to print ultrathin (~5 nm) and smooth silver (Ag) films as the top absorptive reflector and also thick and reflective films as the back reflector. As a result, the printed metal-dielectric-metal FP structures exhibited vivid and saturated colors from blue to green and red on a variety of substrates, including glass, PDMS, and plastics. Importantly, PPD is capable of directly structure writing with a spatial resolution down to 6.5 µm, which is comparable with current colorant based color printing. This new printing technology is expected to have broad use in anti-counterfeit labels, colorimetric sensors, flexible structural color membranes, and decorations. The resolution can be further improved by improving the numerical aperture of the projection system to reduce the beam spot size on the sample surface in future development.

## Results

**Fig. 1:**
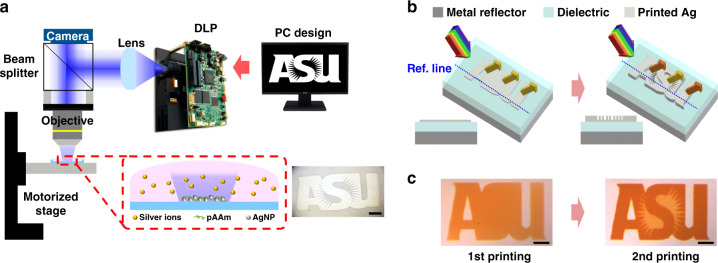
Structural color printing technology. **a** Schematic illustration of the polymer-assisted photochemical deposition (PPD) printing setup and an example of printed ASU logo (scale bar: 500 μm). **b** Three-dimensional scheme showing printing of multilayered film stack into microstructures. **c** Optical images showing an example of printed colored ASU logo corresponding to (**b**). Scale bar: 100 μm.

The metal thin-film deposition was performed at room temperature using our PPD system (Fig. 1a). Ultraviolet (UV) dynamic light projector (DLP) is used as the light source to photochemically induce metal reduction from a precursor solution. Our PPD system has been demonstrated capable of printing different metals, including gold, silver, and platinum^42^. Silver was chosen in this work owing to its high reflectance (>95%) in the visible wavelength range^43^. Here, the precursor contains a metal salt (silver nitrate), reductant (sodium citrate dihydrate), and polymer reactant (pAAm). UV illumination (385 nm) triggers the reduction of silver (Ag) ions to silver nanoparticles (AgNPs), which are then connected into continuous films assisted by the pAAm polymer. Uniquely, this AM system is therefore capable of adjusting both the lateral dimensions of printed metallic structure at the micrometer scale, by selectively turning on and off the digital micro-mirror (DMD) pixels, and also the film thickness at the nanometer level, by controlling the film growth rate and time. For example, the spatial light intensity distribution is controlled by a DMD device within the DLP and flexibly programmed by a computer-generated layout. The light pattern guides the Ag film growth in targeted areas and in designed shapes, exemplified by an Arizona State University (ASU) logo (Fig. 1a insert). The capability of metal printing with precise control in both lateral and vertical dimensions is particularly useful for the creation of an asymmetric FP cavity to simultaneously display multiple structural colors (Fig. 1b). The FP cavity structure consists of an Ag reflector at the bottom, a dielectric spacer in the middle, and a printed Ag thin film on the top. In one example, three different colors can be printed simultaneously, that is a background layer without the top Ag films, a middle ground layer with a very thin (e.g., <10 nm) Ag film, and a foreground layer with slightly thicker (e.g., >10 nm) Ag film. This multi-thickness film stack can be produced in a single print without moving the substrate or refilling the precursor, but rather simply by overlaying two computer-generated image patterns in consecutive illumination steps. For instance, maroon-colored letters of ASU as the foreground can be revealed on and distinguished from the gold-colored middle ground layer (Fig. 1c), creating a high contrast with micrometer scale structural resolution.

**Fig. 2:**
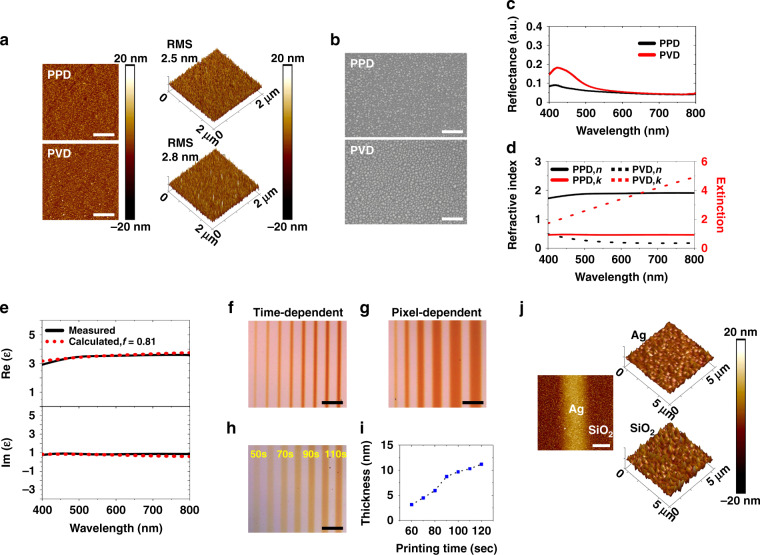
Structural and optical characterization of PPD-printed ultra-thin films. **a**–**c** Surface morphology and optical response of 4-nm-thick Ag films prepared on fused silica substrate by PPD and PVD: **a** 2D (left, scale bar: 500 nm) and 3D (right) profile of AFM images, **b** SEM images (scale bar: 200 nm), and **c** measured relative reflectance. **d** Measured optical properties (*n*, in black; *k*, in red) of 15 nm Ag films prepared on fused silica substrate by PPD (solid lines) and PVD (dot lines), respectively. **e** Measured (black solid line) and calculated (red dotted line) permittivities of PPD film. Top: real part. Bottom: Imaginary part. **f**–**h** Optical images of PPD-printed lines on SiO_2_ (200 nm)/PVD-Ag (85 nm) substrate: **f** single-pixel lines printed from 150 s (left) to 360 s (right), **g** single-pixel (left) to six-pixel (right most) lines printed at 150 s, and **h** four-pixel lines printed from 50 to 110 s. Scale bar: 50 μm. **i** Printed PPD film thickness as a function of printing time extracted from (**h**). **j** Surface morphology of PPD film on SiO_2_ (200 nm)/PVD-Ag (85 nm) substrate. Left: 2D profile of AFM image of 70 s printed four-pixel line from (**h**) (scale bar: 5 μm). Right: 3D profile of AFM images of the printed PPD Ag and sputtered SiO_2_.

To design the FP cavities for desired color printing, we first experimentally investigated the film morphology and optical response of PPD-printed ultra-thin Ag films and compared these to thermally evaporated PVD films (Fig. 2). First, atomic force microscopy (AFM) (Fig. 2a) and scanning electron microscopy (SEM) (Fig. 2b) imaging were used to study the morphology and surface roughness of PPD- and PVD-deposited thin films (both ~4 nm thick on a fused silica substrate, and deposited at a similar rate of 2.4 nm min^−1^). The PPD film was found comparable to but slightly smoother (root-mean-square (RMS) surface roughness 2.5 nm) than PVD film (RMS roughness 2.8 nm). This is attributed to the fact that noble metals such as gold and silver tend to migrate on the surface and nucleate into metal nanoparticles (MNPs), resulting in relatively rough and sometimes discontinuous films when deposited at sub 20 nm by PVD^44,45^. In comparison, reduced MNPs in PPD are stabilized by chemical capping agents and pAAm polymers^46^, which produce continuous thin film even at sub 10 nm thickness. These phenomena were evidently observed by SEM imaging (Fig. 2b). Further, the reflectance spectra of both thin films were examined by a UV-visible (Vis) spectrometer (Fig. 2c). Despite similar reflectance in the longer wavelength range (600–800 nm), PVD film displayed a higher reflectance with a peak at 430 nm compared to PPD film, which was due to the LSPR scattering of AgNPs^47^. These results again confirmed the formation of AgNPs islands rather than continuous structures via PVD deposition. Indeed, the capability of PPD to print ultrathin yet continuous films is much desired in this FP cavity-based color printing demonstration.

To compare the optical refractive indexes (*n*, *k*) of PPD- and PVD-films, 15 nm thick Ag was prepared on fused silica substrates, and examined by UV-near infrared (NIR) spectroscopic ellipsometry (Fig. 2d). Notably, PPD film had a higher refractive index (*n* ~1.90) and lower extinction coefficient (*k* ~0.92) than PVD one in the visible wavelength range (400–800 nm). Clearly, the optical permittivity of aggregated nanoparticles behaved significantly different from ideal metallic films or isolated nanoparticles^48^. This is thought to be due to the presence of polymer (e.g., pAAm has *n* ~1.38, *k* ~0) residue within the printed PPD thin films, effectively forming MNPs/polymer nanocomposite with its optical properties strongly affected by the MNP and polymer material compositions^49,50^. To further investigate the printed PPD film, we employed effective medium theory (EMT) to set up a numerical model that can precisely predict the optical properties of the nanocomposite (details in Supplementary information, Effective medium theory section)^51^. Importantly, the unique optical response of PPD-printed composite film can be successfully interpreted by EMT, evidenced by the fact that EMT-calculated effective permittivity agrees very well with the empirical values with a filling factor of AgNPs fit as 0.81, i.e., 81% of the total volume of nanocomposites (Fig. 2e). This indicated a very high concentration of AgNPs in the nanocomposite matrices.

Then, we studied the impact of printing conditions, particularly printing time and pixel sizes, on the structural lateral resolution and film thickness (Fig. 2f–i). The dielectric-coated metallic substrates, i.e., 200 nm SiO_2_ dielectric layer on 85 nm PVD Ag back reflector, were used in this study. First, the impact of printing time (150 to 360 s) on linewidth was studied using single-pixel straight lines (Fig. 2f). Noticeably, the printing rate on a dielectric-coated metal substrate (6.5 nm min^−1^) was ~2.7 times higher than bare fused silica substrate (2.4 nm min^−1^) (Fig. S1c, d). This is attributed to a ~2.5 times higher electromagnetic field intensity at the printing surface resulting from the reflection of illuminating UV light at the PVD Ag reflector, estimated from FDTD simulation (Fig. S1e). Additionally, the printed lines were found to expand with increased printing time, e.g., 4.6 µm at 150 s (film thickness of 8.6 nm) compared to 8.2 µm at 360 s (thickness 22.5 nm). The observed linewidth broadening is thought attributed to the metal structure nucleation and growth process. It is understood that the reduced MNPs in the solution can go through a diffusion process as printing proceeds, which resulted in wider linewidth. On the other hand, it is also possible the initially printed metal lines would serve as nucleation seeds to guide subsequent metal growth at all directions, including from the line edges, which further enhanced the linewidth increase. Besides the dependence on printing time, the linewidth was also found dependent on pixel sizes (Fig. 2g). Interestingly, single-pixel and two-pixel lines were significantly narrower (4.6 and 7.8 µm, respectively) than larger-pixel (three to six) ones (14 to 24.3 µm), despite the same printing time (150 s). This trend is consistent with our observation of Ag printing on bare fused silica (Fig. S2). In both cases, the metal film thickness (Fig. S2) and film-stack color contrast (Fig. 2g) of printed lines gradually increased with pixel size and reached a plateau with four or more pixels. The correlation between pixel size and film thickness is a unique characteristic of our PPD process. It is attributed to the fact that the metal film growth rate is strongly dependent on the effective electromagnetic field intensity at the nucleation side, which is not only controlled by illumination but also affected by the reflection from just printed metallic thin-film structures. The optical scattering from the rougher edges is expected significantly higher at narrow lines, thus rendering less strong effective electromagnetic field intensity and slower film growth compared to wider lines. Such scattering effect, however, could become less dominant when the linewidth is significantly larger than the illumination wavelength, i.e., 20.3 µm at four pixels compared to 385 nm illumination. Such feature size-dependent printing phenomena bring extra flexibility for PPD to produce multi-colored structures within a single printing process, for example by designing structures of different widths and altering the printing time.

Further, these studies allow calibration of film thickness at different pixel sizes and different printing times for accurate structural design in color printing. For example, four-pixel lines were printed from 50 to 110 s with a 10 s step size on the prepared dielectric (sputtered SiO_2_) on a silver substrate (Fig. 2h). The PPD-printed nanocomposite film thicknesses were examined by AFM and displayed almost linear dependence on the printing time (Fig. 2i). Interestingly, the 3D profile of AFM images also indicated a much smoother surface (RMS = 1.7 nm) for the ultra-thin nanocomposite film (6 nm from 70 s printing) than the SiO_2_/silver substrate itself (RMS = 3.2 nm) (Fig. 2j). This can be understood from the unique PPD printing mechanism that photochemically reduced, pAAm-capped, small AgNPs (estimated 5–10 nm from AFM and SEM) could easily attach to the substrate surface via Van der Waals interactions^42^, thus filling the voids with pAAm and AgNPs and favorably reducing substrate surface roughness^52^.

**Fig. 3:**
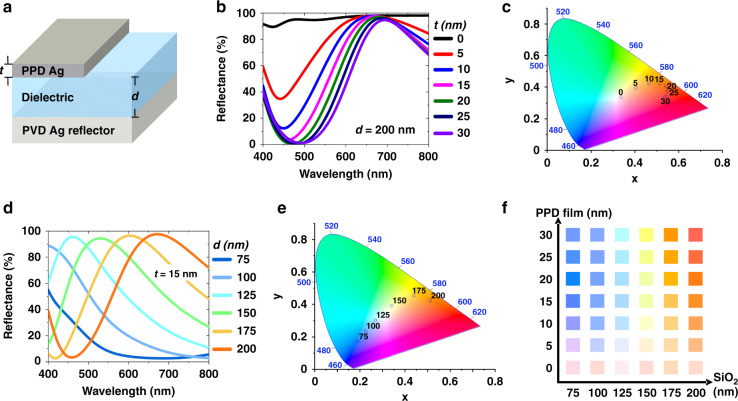
FDTD simulated reflectance spectra for the design of structural colors. **a** Three-dimensional schematic of the FP cavity, with the top-layer PPD-printed thin film thickness *t* and dielectric spacer thickness *d* as variables. **b** Reflectance spectra with variable *t* and fixed *d* = 200 nm. **c** 1931 CIE color coordinates corresponding to (**b**). **d** Reflectance spectra for variable *d* and fixed *t* = 15 nm. **e** CIE color coordinates corresponding to (**d**). **f** The color palettes are based on the simulated reflectance spectra with varying *d* and *t*.

To guide our color printing design, FDTD simulations were conducted with empirically measured optical indexes of nanocomposite and dielectric (SiO_2_) films to understand their thickness impact on the color display. We started with an investigation into the impact of the material effect of top metal film materials, i.e. PPD-printed or PVD-deposited, using the same cavity structure, consisting of 85 nm PVD Ag back reflector at the bottom, 200 nm SiO_2_ dielectric layer in the middle and a variable metal film thickness *t* (0 to 30 nm, 5 nm step size) on the top (Fig. 3a), was used in this study. Here the change in the thickness of the PPD-printed nanocomposite film directly affects both the reflection (and according to the transmission into the FP cavity) and also the phase accumulation (Fig. S3). Therefore, the film thickness can strongly modulate the interference effects in the FP cavity, shifting both the amplitude and resonance of reflected light (Fig. 3b). Using 15 nm PPD film as a top layer, significantly broaden reflectance spectra (~300 nm at full-width half maximum (FWHM)) and relatively high reflectance (98% at 670 nm) were calculated in the visible wavelength range (Fig. 3b ‒ solid pink line), which facilitated highly-contrast and bright color printing in selected wavelength ranges^17^. As aforementioned, PPD film effectively acted as an absorptive composite material composed of highly concentrated absorbing AgNPs^53^ and non-absorbing pAAm polymer^54^. This behavior differs significantly from using PVD Ag films as the top layer of the FP cavity (Fig. S4), which was calculated to display a high reflectance with a much narrower resonance in the visible wavelength range and much less effective to produce saturated colors. In comparison, the predicted equivalent colors from our simulated reflectance spectra were illustrated on the 1931 International Commission on Illumination (CIE) chromaticity diagram by using colorimetric transformations (Fig. 3c for PPD top film and Fig. S4c for PVD film), clearly showing a much broader range of color tuning by PPD printing compared to PVD film that barely displayed color differences by changing the film thickness.

Similarly, we conducted an FDTD simulation with various dielectric layer thicknesses (*d*) to obtain a wider color gamut (Fig. 3d, e). The cavity structure was composed of an 85 nm PVD Ag back reflector, 75 to 200 nm SiO_2_ dielectric spacer (*d*, 25 nm step size) in the middle, and a 15 nm PPD film on the top. As the SiO_2_ thickness *d* increases, the resonance peak wavelength redshifted from 330 to 400, 460, 530, 600, and 670 nm, respectively (Fig. 3d). Compared to the color tuning effect of PPD-printed metal film thickness (Fig. 3b), the dielectric layer spacer has a more pronounced impact in modulating the reflectance amplitude and resonance peak wavelengths. The predicted colors corresponding to the reflectance spectra on the CIE chromaticity diagram (Fig. 3e) further clearly indicated a broader color gamut than that with varying PPD film thickness *t* (Fig. 3c). This can be understood as the result of significant change in the optical path and accumulated phase in the cavity^16–18^. To visualize the printed colors, a two-dimensional color palette was created with varying SiO_2_ dielectric layers and the top PPD film thicknesses (Fig. 3f). For example, the palette showed highly saturated vivid colors of blue, light blue, cyan, light green, orange, and blood orange, respectively, as *d* increases while keeping *t* = 20 nm. Noticeably, distinguishable colors were also obtained by simply modifying *t* with the same dielectric layer, which provides more degrees of freedom for tunable color printing.

**Fig. 4:**
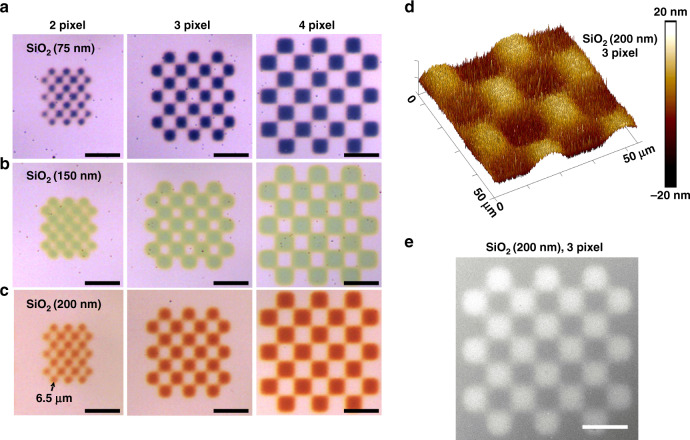
Experimental demonstrations of color printing with micrometer resolution. **a**–**c** Optical images of various pixel-sized checkerboards printed on the SiO_2_/PVD Ag substrate with the SiO_2_ thickness as 75, 150, and 200 nm, respectively. Scale bar: 30 μm. **d** AFM image and **e** SEM image (scale bar: 30 μm) of the printed three pixel-checkerboard on 200 nm SiO_2_ layer. The PPD film was ~20 nm thick.

Based on the above simulation results, we employed various thicknesses of SiO_2_ dielectric layer (*d* = 75, 150, and 200 nm, respectively) on the 85 nm PVD Ag back reflector to experimentally demonstrate the color generation (Fig. 4). The printing was carried out for 180 s (thickness ~20 nm) with checkerboard patterns to characterize the spatial resolution of our PPD system for color printing. The experimentally produced colors were consistent with the simulation-predicted color palette with a spatial resolution down to 6.5 µm (Fig. 4a–c). The 3D profile of the AFM image again confirmed a much smoother surface (RMS roughness 1.5 nm) of PPD film than sputtered SiO_2_ dielectric layer (RMS roughness 3.2 nm) with sufficiently distinguishable microscale features (Fig. 4d). Interestingly, distinct structures were well preserved even at the corners and boundaries of neighboring areas (Fig. 4a–c), indicating the printing faithfully produced microstructured patterns as confirmed by the SEM image (Fig. 4e). This demonstration implies that our PPD-based AM technology distinguishes itself from conventional structural color printing methods in its capability of creating micro-scaled colored features without any photolithography processes.

**Fig. 5:**
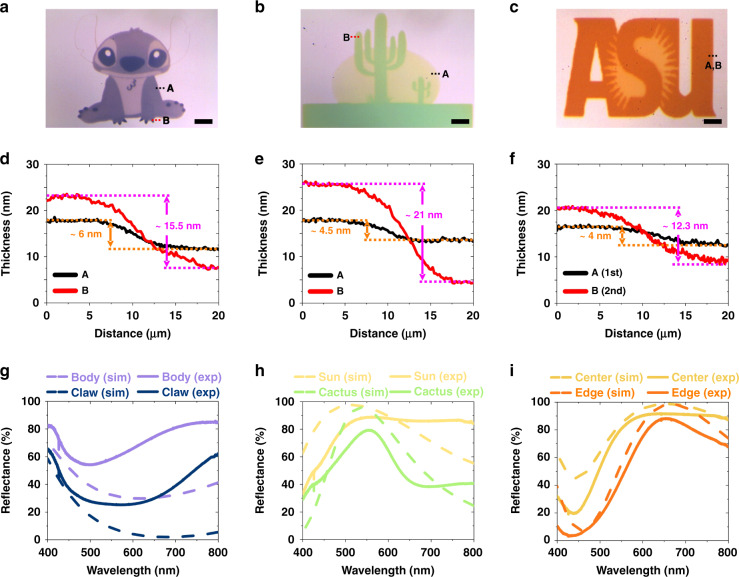
Demonstration of the feasibility of printing complex structures with various colors. **a** A cartoon character “Stitch” (*d* = 75 nm), **b** a symbol of Arizona “Cactus” (*d* = 150 nm), and **c** a logo of “ASU” (*d* = 200 nm). Scale bar: 100 μm. **d**–**f** AFM measured PPD film thicknesses *t* for each structure corresponding to (**a**–**c**), respectively. **g**–**i** FDTD simulated reflectance spectra (dashed lines) and experimentally measured reflectance spectra (solid lines) for each structure corresponding to (**a**–**c**), respectively.

To investigate the feasibility of color printing of complex structures, three different images were used on dielectric-coated silver substrates (Fig. 5). Given the layer-by-layer nature of the PPD process, those images were divided into several layers for printing. A colored microscale cartoon image of “Stitch” was printed on a 75 nm SiO_2_ dielectric layer to represent blue color over an area of 670 µm × 610 µm (Fig. 5a). A multistep printing, i.e., 100 s for belly and ears, 150 s for head, arms, and legs, and 210 s for eyes, nose, and claws, was found to achieve the best color contrast. Besides, a “Cactus”, the symbol of Arizona state, was printed over an 860 µm × 620 µm area on a 150 nm SiO_2_ dielectric layer as the representative for green color (Fig. 5b). To obtain the best printing quality, the printing time was set as 45 s for sun and 210 s for cactus, respectively. Additionally, a colored microscale logo of “ASU” was printed over an area of 720 µm × 450 µm on a 200 nm SiO_2_ dielectric layer to demonstrate red and orange color printing (Fig. 5c). The printing time was 60 s for the ASU logo background and 180 s for ASU letters, respectively. Our AFM analysis (Fig. 5d‒f) further confirmed the highly saturated and high-contrast colors were created from as thin as 4 nm PPD films. Lastly, FDTD simulations were conducted with the empirically measured film thicknesses and refractive indexes to calculate the reflectance spectra and compared to the spectra measured by UV-Vis spectrometer (Fig. 5g‒i). Noticeably, the measured reflectance spectra (solid lines) showed large modulation in the reflectance similar to simulated spectra (dashed lines), despite small discrepancies possibly attributed to differences in film geometries and material properties between simulations and experiments. We also investigated the effect of polymer encapsulation layer on color retention time using the same structures (details in Supplementary information, Color retention time tests section). Although the reflectance spectra was slightly red-shifted after 14 days of printing (Fig. S5d) possibly due to additional reaction between AgNPs and poly(methyl methacrylate) (PMMA), the encapsulation layer significantly improved the color retention time of the printed image (Fig. S5b). Nevertheless, these results convincingly demonstrate that the PPD-based structural color printing is an alternative and cost-effective solution for high-quality and micro-scaled color printing applications. In future studies, it is possible to print multiple colors by engineering the FP-cavity dielectric layers at different thicknesses on one sample. AM technology also allows photo-initiated polymerization that can be used to print dielectric spacers at designed thicknesses^55^.

**Fig. 6:**
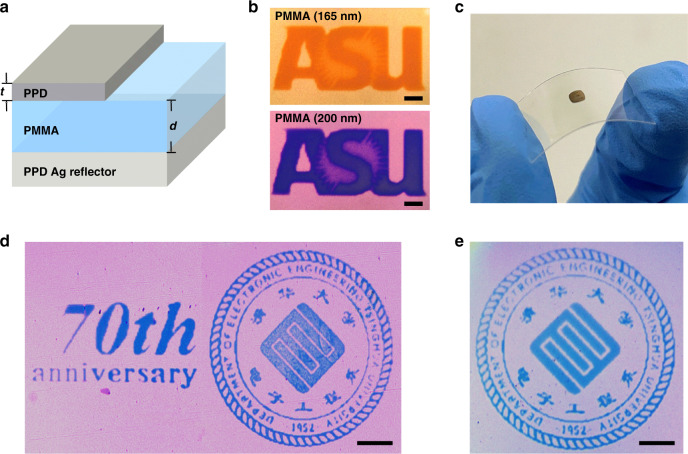
Photolithography and vacuum-free structural color printing. **a** Three-dimensional schematic of the FP cavity. **b** An exhibited colored ASU logo on the dielectric-coated metallic layer with various PMMA thicknesses using fused silica as a substrate. Scale bar: 50 μm. **c** Camera image of fabricated FP cavity on PET substrate with slight bending capability. **d**, **e** Optical images of produced colored DEETHU logo on the dielectric-coated metallic layer using PET and PDMS as substrates, respectively. Scale bar: 100 μm.

The above color printing demonstrations were achieved without photolithography, yet they still required several vacuum deposition processes for substrate preparation. To eliminate any vacuum depositions altogether, we further utilized PPD films to print both the bottom metal reflector and top-layer thin film and used spin-coated PMMA as a dielectric layer (Fig. 6a). The reflectance from a back reflector strongly influences the FP cavity optical performance and directly affects the color saturation^56^. Here we examined the PPD-printed back reflector on the fused silica substrates after 8, 9, and 10 min printing by UV-Vis spectrometer, using a protected silver mirror as a reference (Fig. S6a). Interestingly, 8 min printed PPD film showed the highest relative reflectance (79% at 600 nm) compared to longer time (9 and 10 min, with 77 and 74%, respectively) printed films, possibly attributed to increased surface roughness (Fig. S6b) due to undesired particle growth and resultant undesired scattering loss^50^. Therefore, 8 min printed PPD back reflector was chosen for our printing on different substrate materials. The PPD back reflector was characterized by a surface profiler, UV-Vis spectrometer, AFM, and SEM. Remarkably, the film surface was smoother (RMS roughness 3.5 nm) than the PVD back reflector (RMS roughness 5.5 nm) at the same thickness (85 nm) (Fig. S7a‒c), despite lower reflectance (Fig. S7d). Then PMMA was spin-coated on the back reflector, with its thickness adjusted by changing spin coating speed. The PMMA refractive index was characterized by ellipsometry and found similar to that of SiO_2_ in the visible wavelength range (Fig. S8). The PMMA was solidified overnight and briefly treated by oxygen plasma prior to the top-layer metal printing. A microscale logo of ‘ASU’ with a size of 410 µm × 160 µm was printed on 165 nm- and 200 nm-thick PMMA layers (Fig. 6b), producing different colors with a spatial resolution of ~7 µm. Both the color saturation and printing resolution were comparable to using PVD-deposited back reflectors. A lower contrast was observed, possibly because of the lower reflectance of a back reflector, which could negatively affect the optical reflectance from the FP cavity.

Printing on flexible substrate is desired in many color printing applications, yet it could be problematic when using conventional fabrication processes (e.g., PVD and photolithography) due to the instability of the substrates when exposed to vacuum, heat, or organic solvent environments^57,58^. A unique advantage of PPD printing is its room temperature, ambient, and solvent-free printing process that allows it to be compatible with various substrates, as demonstrated in our previous work^40^. Here we further demonstrate micro-scaled structural color printing onto flexible PET and PDMS, the most well-known flexible substrates. We printed the logo of the department of electronic engineering of Tsinghua University (DEETHU) in order to celebrate their 70th anniversary, which is the theme of this special issue. Clearly, the FP cavity was well fabricated on PET and intact with a slight bending test (Fig. 6c). The optical images of the colored DEETHU logo on PET and PDMS substrates (Fig. 6d, e), respectively, strongly demonstrated the feasibility of color printing with a spatial resolution of about 6.3 µm, completely comparable to our printing on rigid silica substrates.

## Discussion

In summary, we have developed a new approach to print micro-scaled structural colors using room temperature, ambient, and low-cost PPD process. The ultra-thin PPD-printed film showed satisfactory surface morphology characteristics in surface roughness and continuity and acted as an absorptive layer in a FP cavity structure. The FP cavity designs were optimized by coupling experimental studies and FDTD simulations to modulate the reflectance in the visible wavelength range and to produce highly saturated and bright colors. We have further demonstrated the printing of complex structures in logos and pictures of different colors with a spatial resolution down to 6.5 µm, on both rigid fused silica and flexible substrates. Our demonstrations show that the PPD color printing technology can eliminate complex photolithography and vacuum deposition processes, thus opening new windows to a wide variety of applications including colorimetric sensors, surface decoration, wearable optical devices, and flexible display with high resolution and low cost.

## Materials and methods

### Materials

Silver nitrate (ACS reagent, ≥99.0%), sodium citrate dihydrate (≥99.0%), allylamine (AAm, ≥99.0%), 2-hydroxy-4′-(2-hydroxyethoxy)-2-methylpropiophenone (Irgacure 2959, 98%) and trichloro(1*H*,1*H*,2*H*,2*H*-perfluorooctyl)silane were purchased from Sigma-Aldrich. PMMA (950 K A4) was purchased from MicroChem. Dow Corning Sylgard 184 silicone elastomer was purchased from VWR. Polyethylene terephthalate (PET) film (PF-40/1.5-X4) was acquired from Gel-Pak. DLP^®^ LightCrafter™ E4500 MKII™ UV (385 nm, 2 W) was purchased from EKB Technologies Ltd. The motorized stage (100 mm motorized linear translation stage, stepper motor, 1/4″-20 taps) and CMOS camera (1280 × 1024 pixels) were purchased from Thorlabs. All chemicals were used as received without further purification.

### Preparation of Ag printing precursor

The Ag printing precursor was prepared by mixing silver nitrate, sodium citrate dihydrate, and poly(allylamine) (pAAm). Silver nitrate and sodium citrate dihydrate solid powders were dissolved in DI water respectively, to obtain a 100 mM stock solution for each chemical. pAAm stock solution was prepared by photo-polymerization of AAm. About 200 µL AAm stock solution which contained 1 M AAm and 0.57 mg mL^−1^ of Irgacure 2959 was illuminated under UV light (BlueWave^®^ 200 UV curing spot lamp, 365 nm, 3.0 W cm^−2^) for 15 min. The polymerized solution was used as it is. To produce Ag printing precursor, 80 µL silver nitrate stock solution, 60 µL sodium citrate dihydrate stock solution, and 16 µL pAAm stock solution were added into 844 µL DI water. The prepared precursor should be consumed immediately.

### Preparation of substrates

Bare Si wafer was used as a substrate to prepare ultra-smooth polydimethylsiloxane (PDMS) sheets. Self-assembled monolayers (SAMs) were formed on the substrate surface by using trichloro(1*H*,1*H*,2*H*,2*H*-perfluorooctyl)silane at 100 °C for 30 min as an anti-sticking layer followed by solvent and RCA-1 cleaning. After cooling the Si substrate, a mixture of Sylgard 184 silicone elastomer and its curing agent (10:1 wt/wt) was poured onto it, degassed, and further cured at 70 °C for 3 h. The prepared PDMS was gently peeled off from the substrate, cut into rectangles, and transferred onto solvent cleaned coverslip for easy handling during the printing process. The PET substrate was delaminated from commercially available Gel-film and used as it is. Fused silica substrate was solvent and RCA-1 cleaned to remove absorbed organic and inorganic contaminants. All the substrates were nitrogen blown and cleaned with an oxygen plasma cleaning system (Tergeo plasma cleaner, 75 W, 5 sccm) for 30 s prior to the further fabrication process.

### Ag and SiO_2_ film deposition

Sub-5 nm Ag film was deposited onto fused silica substrate by thermal evaporator (Denton Benchtop turbo) to compare the quality of ultra-thin printed PPD Ag film with conventionally evaporated PVD one. Ultra-pure silver pellet (99.999% purity, Materion) was used with a deposition rate of 0.4 Å s^−1^ which is identical to the printed Ag deposition rate with the same thickness. This deposition was carried out without an adhesion layer for a precise comparison to printed Ag film. Metal/dielectric substrate was prepared using a conventional deposition process to demonstrate the feasibility of structural color printing using PPD. Three-nanometer chromium was evaporated as an adhesion layer, then 85 nm Ag film was deposited as a back reflector onto fused silica substrate at 1.7 Å s^−1^ with the same evaporator. The deposition rate was identical to printed Ag film with the same thickness. SiO_2_ dielectric layer was deposited at 0.5 Å s^−1^ using the radio frequency (RF) sputtering system (Kurt J. Lesker) with various thicknesses to adjust the resonance peak of the cavity.

### FP cavity-based structural color printing using PPD

The aforementioned metal/dielectric substrate was used for the demonstration of structural color printing. Desired printing patterns were designed in software Paint due to its pixel-based interface. A reservoir was made by PDMS in the same manner as described in the preparation of substrates section was placed onto the substrate, then filled with the printing precursor. Designed patterns were illuminated by digital mirrors in the DLP^®^ LightCrafter™ E4500 MKII™ system from the top through the precursor using a 10x objective lens with a numerical aperture (NA) of 0.3 to print Ag on the substrate surface (Fig. 1a). The sample height was precisely controlled by a motorized stage to achieve the best printing quality. The illuminated patterns were dynamically changed by PC software after a certain amount of time for the first layer printing. After the completion of printing, the reservoir was removed from the substrate, and the substrate was rinsed with DI water and dried with nitrogen blow. To prove photolithography and vacuum deposition-free structural color printing on rigid and flexible substrates, fused silica, PDMS, and PET substrates were used. To begin with, 85 nm thick PPD Ag film was printed as a back reflector, then PMMA was spin-coated onto printed film as a dielectric layer. The substrates were left at room temperature for overnight to evaporate the solvent and solidify PMMA. Oxygen plasma treatment (Tergeo plasma cleaner, 25 W, 10 sccm) was employed for 30 s to make the PMMA surface be hydrophilic, and the printing surface became more attractive to the printing precursor. Finally, designed patterns were printed on the top of the PMMA surface in the same manner to complete structural color printing.

### Film characterization

Atomic force microscopy (AFM, Bruker Multimode 8) was used to examine the thickness and surface roughness of both PPD and PVD films. A tapping mode was employed at the ambient condition with various scan sizes and at a scan rate of 1 Hz. The detailed surface morphology was inspected by scanning electron microscopy (SEM, Hitachi S-4700 FESEM) with an acceleration voltage of 5 keV and a current of 10 µA. A thin layer of gold/palladium was sputtered (Cressington sputter coater 108) on the samples to enhance imaging resolution prior to SEM measurements. Optical properties (refractive index *n*, extinction coefficient *k*) of PPD and PVD films and dielectric layers were measured by UV-NIR spectroscopic ellipsometry (J.A. Woollam, M-2000). Olympus BX53 fluorescent microscope coupled Horiba iHR320 imaging spectrometer was utilized to record all the optical images and reflectance spectra of fabricated samples. A protected silver mirror (Thorlabs, PF10-03-P01) was used as a reference to calculate the relative reflectance of the samples, which has optical reflectance over 97.5% in the visible wavelength range.

### Simulation

The finite-difference time-domain (FDTD) simulations were carried out to calculate reflectance spectra in the visible wavelength range. Various thickness of printed PPD film and SiO_2_ dielectric layer were employed to design the structural geometry of the FP cavity for structural color printing. Periodic boundary conditions (±*x*, ±*y* direction) and perfectly matched layers (±*z* direction, parallel to the propagation of electromagnetic waves) were used within a unit cell of 200 nm along the ±*x* and ±*y* direction. The unit cell consisted of an 85 nm thick PVD back reflector, *d* nm SiO_2_ dielectric layer, and *t* nm PPD or PVD Ag films. The plane wave in the visible wavelength range (400–800 nm) was used as the light source, which was incident perpendicular to the unit cell. The mesh sizes were set as 2 nm along the ±*x* and ±*y* direction and 1 nm along the ±*z* direction. The measured optical properties (*n*, *k*) of each material were used for simulation. The reflectance was monitored in the x–y plane, ∼1000 nm away from the top surface of a structure. Using colorimetric transformations, the simulated reflectance spectra were mapped on the CIE 1931 chromaticity diagram to display the corresponding colors. They were compared with experimental observations.

### Effective medium theory

Effective medium theory (EMT) was utilized to numerically calculate the effective permittivity (*ε*_*eff*_) of the printed PPD film to characterize its optical properties. Bruggeman’s EMT model was employed for calculation due to the aggregated structure of AgNPs and pAAm in the nanocomposites. The formula for *ε*_*eff*_ can be expressed as $\varepsilon _{eff} = \varepsilon _p\left[ {1 + \frac{{f(\varepsilon _{np} - \varepsilon _p)}}{{\varepsilon _p + n(1 - f)(\varepsilon _{np} - \varepsilon _p)}}} \right]$, where *ε*_*np*_ and *ε*_*p*_ were the permittivities of AgNPs and pAAm, respectively, and *f* was the filling factor of AgNPs in the nanocomposite. A shape factor *n* was introduced as a fitting parameter to generalize the equation. To investigate the average AgNP size, we performed pAAm concentration-dependent PPD printing. From the SEM images with various pAAm concentrations (Fig. S11), we observed particles from sub-5 to 10 nm with distinguishable contrast, particularly in 30 mM of pAAm concentration, which is probably attributed to higher pAAm capping efficiency for AgNPs (Fig. S11e). Therefore, the average size of AgNPs was set as 10 nm in diameter for the purpose of calculating *ε*_*np*_ using our model.

## Supplementary information

Supplementary information for Structural color printing via polymer-assisted photochemical deposition

## References

[1] Wang H (2017). Full color generation using silver tandem nanodisks. ACS Nano.

[2] Kumar K (2012). Printing colour at the optical diffraction limit. Nat. Nanotechnol..

[3] Hail CU (2020). A plasmonic painter’s method of color mixing for a continuous red-green-blue palette. ACS Nano.

[4] Liu X, Huang Z, Zang JF (2020). All-dielectric silicon nanoring metasurface for full-color printing. Nano Lett..

[5] Kim M (2018). Active color control in a metasurface by polarization rotation. Appl. Sci..

[6] Huang Y (2020). Polarization-controlled bifunctional metasurface for structural color printing and beam deflection. Opt. Lett..

[7] Nam H (2016). Inkjet printing based mono-layered photonic crystal patterning for anti-counterfeiting structural colors. Sci. Rep..

[8] Chen K (2017). Multicolor printing using electric-field-responsive and photocurable photonic crystals. Adv. Funct. Mater..

[9] Yakovlev AV (2016). Inkjet color printing by interference nanostructures. ACS Nano.

[10] Rana AS (2020). Engineering the absorption spectra of thin film multilayer absorbers for enhanced color purity in CMY color filters. Optical Mater. Express.

[11] Yakovlev AV (2016). Inkjet printing of TiO_2_/AlOOH heterostructures for the formation of interference color images with high optical visibility. Sci. Rep..

[12] Nagasaki Y, Suzuki M, Takahara J (2017). All-dielectric dual-color pixel with subwavelength resolution. Nano Lett..

[13] Yang WH (2020). All-dielectric metasurface for high-performance structural color. Nat. Commun..

[14] Sun S (2017). All-dielectric full-color printing with TiO_2_ metasurfaces. ACS Nano.

[15] Hodgson, N. & Weber, H. in *Laser Resonators and Beam Propagation* 2nd edn (eds Hodgson, N. & Weber, H.) Ch. 4 (Springer, 2005).

[16] Yang ZM (2016). Reflective color filters and monolithic color printing based on asymmetric Fabry-Perot cavities using nickel as a broadband absorber. Adv. Optical Mater..

[17] Lee J, Kim J, Lee M (2020). High-purity reflective color filters based on thin film cavities embedded with an ultrathin Ge_2_Sb_2_Te_5_ absorption layer. Nanoscale Adv..

[18] Yang ZM (2017). Microscopic interference full-color printing using grayscale-patterned Fabry-Perot resonance cavities. Adv. Optical Mater..

[19] Kim SJ (2019). Solution-processable nanocrystal-based broadband Fabry-Perot absorber for reflective vivid color generation. ACS Appl. Mater. Interfaces.

[20] Lee IH (2019). Selective photonic printing based on anisotropic Fabry-Perot resonators for dual-image holography and anti-counterfeiting. Opt. Express.

[21] Wang YS (2019). Fabrication of Fabry-Perot-cavity-based monolithic full-color filter arrays using a template-confined micro-reflow process. J. Micromech. Microeng..

[22] Hedayati MK, Elbahri M (2017). Review of metasurface plasmonic structural color. Plasmonics.

[23] Yang B (2019). Structural colors in metasurfaces: principle, design and applications. Mater. Chem. Front..

[24] Furukawa S, Masui T, Imanaka N (2006). Synthesis of new environment-friendly yellow pigments. J. Alloy. Compd..

[25] Fang YC (2021). Eco-friendly colorization of textile originating from polydopamine nanofilm structural color with high colorfastness. J. Clean. Prod..

[26] Li ZB, Clark AW, Cooper JM (2016). Dual color plasmonic pixels create a polarization controlled nano color palette. ACS Nano.

[27] Xu T (2010). Plasmonic nanoresonators for high-resolution colour filtering and spectral imaging. Nat. Commun..

[28] Shrestha VR (2015). Polarization-tuned dynamic color filters incorporating a dielectric-loaded aluminum nanowire array. Sci. Rep..

[29] Lim KTP (2019). Holographic colour prints for enhanced optical security by combined phase and amplitude control. Nat. Commun..

[30] Zhang F (2020). Simultaneous full-color printing and holography enabled by centimeter-scale plasmonic metasurfaces. Adv. Sci..

[31] Kim DY (2019). Electroactive soft photonic devices for the synesthetic perception of color and sound. Adv. Mater..

[32] Banisadr S, Oyefusi A, Chen J (2019). A versatile strategy for transparent stimuli-responsive interference coloration. ACS Appl. Mater. Interfaces.

[33] Wu YKR (2013). Angle-insensitive structural colours based on metallic nanocavities and coloured pixels beyond the diffraction limit. Sci. Rep..

[34] Ellenbogen T, Seo K, Crozier KB (2012). Chromatic plasmonic polarizers for active visible color filtering and polarimetry. Nano Lett..

[35] Odintsova GV (2019). High-resolution large-scale plasmonic laser color printing for jewelry applications. Optical Express.

[36] Rezaei SD (2019). Tunable, cost-effective, and scalable structural colors for sensing and consumer products. Adv. Optical Mater..

[37] Kenanakis G (2017). Perfect absorbers based on metal-insulator-metal structures in the visible region: a simple approach for practical applications. Appl. Phys. A.

[38] Li ZY, Butun S, Aydin K (2015). Large-area, lithography-free super absorbers and color filters at visible frequencies using ultrathin metallic films. ACS Photonics.

[39] Kajtár G (2016). Theoretical model of homogeneous metal-insulator-metal perfect multi-band absorbers for the visible spectrum. J. Phys. D: Appl. Phys..

[40] Kats MA (2013). Enhancement of absorption and color contrast in ultra-thin highly absorbing optical images. Appl. Phys. Lett..

[41] Kats MA, Capasso F (2016). Optical absorbers based on strong interference in ultra-thin films. Laser Photonics Rev..

[42] Zhao Z (2020). Printing continuous metal structures via polymer-assisted photochemical deposition. Mater. Today.

[43] Barrie JD (2011). Control of stress in protected silver mirrors prepared by plasma beam sputtering. Appl. Opt..

[44] Jeong E (2020). Minimizing optical loss in ultrathin Ag films based on Ge wetting layer: insights on Ge-mediated Ag growth. Appl. Surf. Sci..

[45] Chen WQ (2010). Ultra-thin ultra-smooth and low-loss silver films on a germanium wetting layer. Opt. Express.

[46] Jiran E, Thompson CV (1990). Capillary instabilities in thin films. J. Electron. Mater..

[47] Barman B (2017). Formation of plasmonic silver nanoparticles using rapid thermal annealing at low temperature and study in reflectance reduction of Si surface. Adv. Nat. Sci. Nanosci. Nanotechnol..

[48] Reshetnyak VY (2018). Effective medium theory for anisotropic media with plasmonic core-shell nanoparticle inclusions. Eur. Phys. J..

[49] Vodnik V (2012). Silver/polystyrene nanocomposites: optical and thermal properties. Polym. Compos..

[50] Al-Ramadhan ZA, Salman JA, Humd HAK (2016). Optical and morphological properties of (PVA-PVP-Ag) nanocomposites. Int. J. Sci. Res..

[51] Cai, W. S. & Shalaev, V. *Optical Metamaterials: Fundamentals and Applications* (Springer, 2010).

[52] Albaladejo S, Marqués MI, Sáenz JJ (2011). Light control of silver nanoparticle’s diffusion. Opt. Express.

[53] Dahmouchène N (2008). Silver nanoparticles embedded in polymer matrices-a FTIR-SE study. Phys. Status Solidi (c.).

[54] Wankhede YB (2013). Synthesis and characterization of silver nanoparticles embedded in polyaniline nanocomposite. Adv. Mater. Lett..

[55] Li WD (2020). Stereolithography based additive manufacturing of high-*k* polymer matrix composites facilitated by thermal plasma processed barium titanate microspheres. Mater. Des..

[56] Jang J (2020). Spectral modulation through the hybridization of Mie-scatterers and quasi-guided mode resonances: realizing full and gradients of structural color. ACS Nano.

[57] Yu Y, Yan C, Zheng ZJ (2014). Polymer-assisted metal deposition (PAMD): a full-solution strategy for flexible, stretchable, compressible, and wearable metal conductors. Adv. Mater..

[58] Chou N, Jeong J, Kim S (2013). Crack-free and reliable lithographical patterning methods on PDMS substrate. J. Micromech. Microeng..

