# Supplementary material for: Structural color printing via polymer-assisted photochemical deposition
Source: Light Sci Appl. 2022 Apr 6;11:84. doi: 10.1038/s41377-022-00776-x (PMC8986859; doi:10.1038/s41377-022-00776-x)
Supplement: Supplementary file 1 — Supplementary information for Structural color printing via polymer-assisted photochemical deposition [file 41377_2022_776_MOESM1_ESM.pdf]

Supplementary information for Structural color printing via polymer-assisted photochemical deposition

Shinhyuk Choi[1]*, Zhi Zhao*[1,2,3]*, Jiawei Zuo*[1]*, Hossain Mansur Resalat Faruque*[1]*, Yu Yao*[1] *and Chao Wang*[1,2,*]

[1]*School of Electrical, Computer and Energy Engineering, Arizona State University, Tempe, AZ 85287, USA*

[2]*Center for Molecular Design and Biomimetics at the Biodesign Institute, Arizona State University, AZ 85287, USA*

[3]*College of Materials Science and Engineering, Key Laboratory of Advanced Functional Materials, Education Ministry of China, Beijing University of Technology, Beijing, 100124, China*

[*] Corresponding author.

E-mail address: wangch@asu.edu (C. Wang)

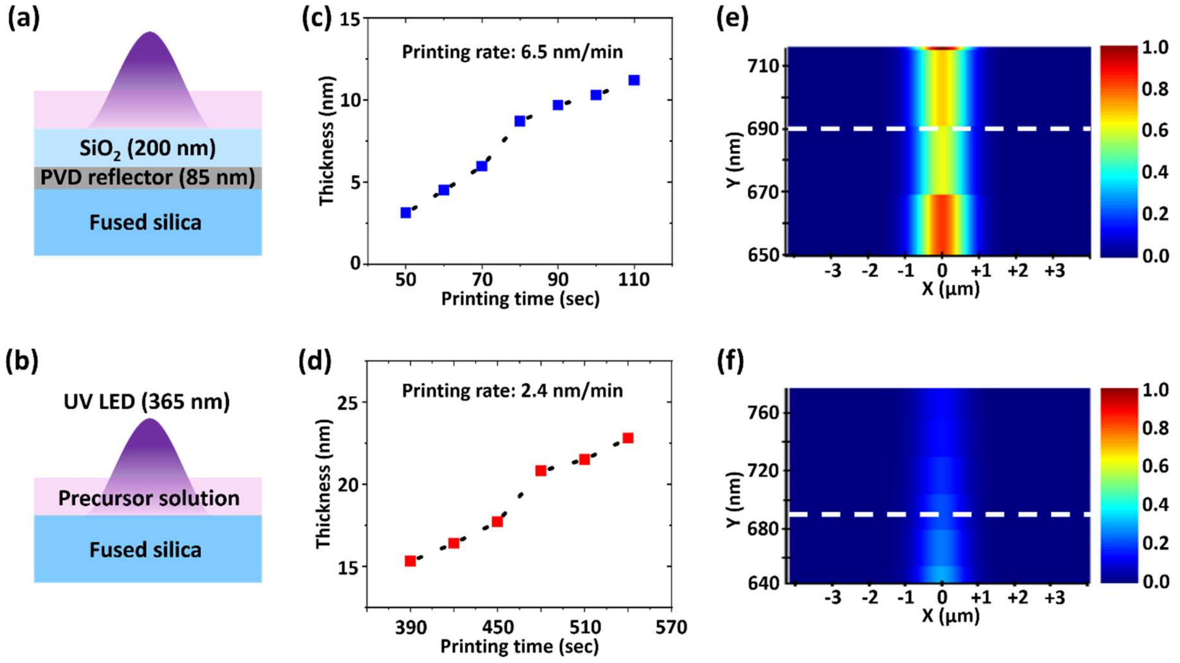

Figure S1. Structure dependent printing behavior. Schematic cross-section view of printing substrate structures (a) with the SiO_2/PVD Ag reflector and (b) without the reflector. (c and d) Printed PPD film thickness as a function of printing time corresponding to (a) and (b), respectively. (e and f) FDTD simulated the electromagnetic field intensity distribution in corresponding to (a) and (b), respectively. White dashed line indicates the printing surface.

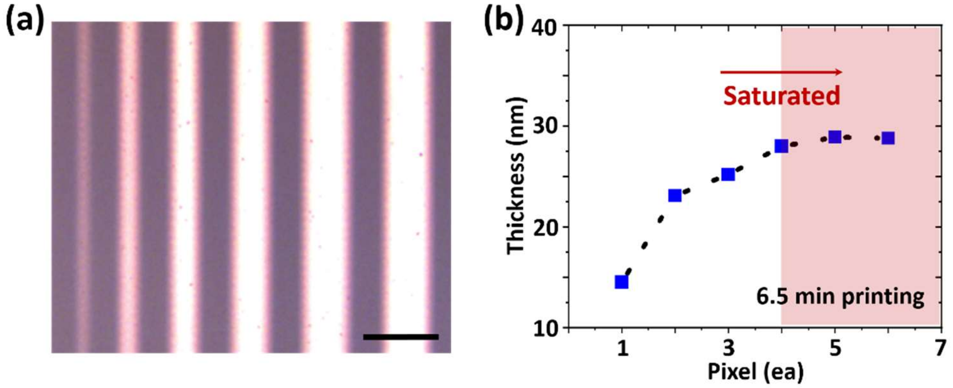

Figure S2. Characterization of the printed PPD film. (a) Pixel-dependent PPD printing on the fused silica substrate. Scale bar: 50 μm. (b) Printed PPD film thickness as a function of pixel size extracted from (a). PPD film thickness saturated after 4 pixel.

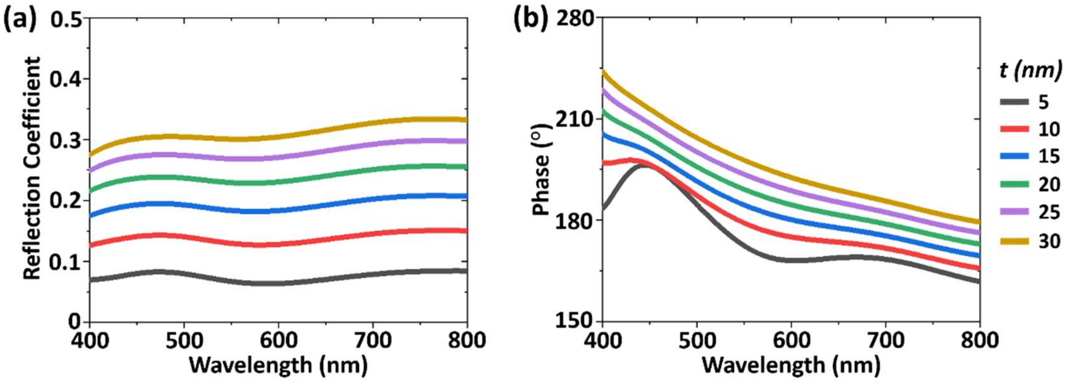

Figure S3. FDTD simulation of the printed PPD film. Thickness-dependent (*t*) (a) reflection coefficient of PPD film and (b) phase shift accumulated at the PPD and fused silica interface.

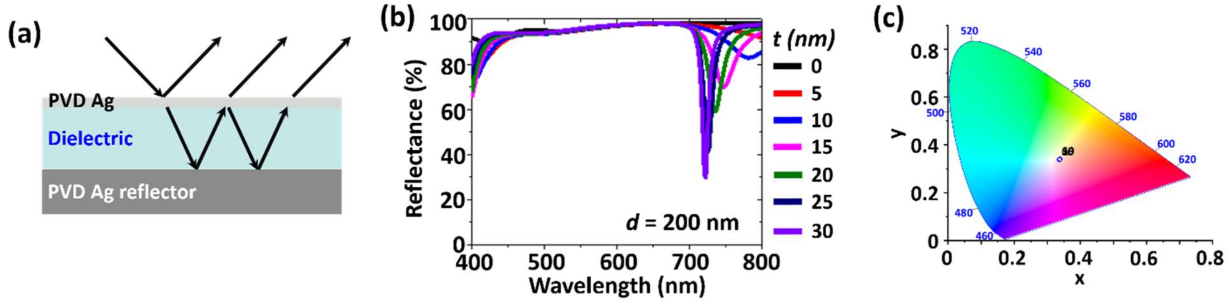

Figure S4. FDTD simulated reflectance spectra for structural colors. (a) Basic configuration of an interference effect in the FP cavity. (b) Reflectance spectra according to the top PVD film thickness with fixed dielectric thickness (200 nm). (c) 1931 CIE color coordinates corresponding to (b).

Color retention time tests

The color retention time was also investigated by following experiments. Briefly, a 'Cactus' was printed on two dielectric-coated silver substrates with exactly the same film thicknesses (150 nm sputter-deposited SiO_2 on 85 nm evaporated Ag), then 50 nm PMMA was spin coated on the one of them as an encapsulation layer. The microscopic images and reflectance spectra were taken immediately after color printing and after 14 days, respectively. The samples were stored in ambient condition during the whole experiments. The sample without an encapsulation layer showed a slight faded color after 14 days (Fig. S5a). From the reflectance spectra, the smaller modulation depth in the visible wavelength was found after 14 days due to oxidation of printed silver, which corresponded to less vivid color (Fig. S5c). On the other hand, the sample with an encapsulation layer still exhibited vivid color after 14 days in ambient condition (Fig. S5b). Although the reflectance spectra indicated a slight red-shift, probably due to the additional reaction

between AgNPs and PMMA[1], the modulation depth was observed to be similar as the pristine color, which implied that the color was well preserved by an encapsulation layer. Interestingly, the spin coated PMMA layer not only acted as encapsulation layer, but also increased color saturation behavior. Further investigation for the impact of various encapsulation layers on the color saturation and retention time will be studied.

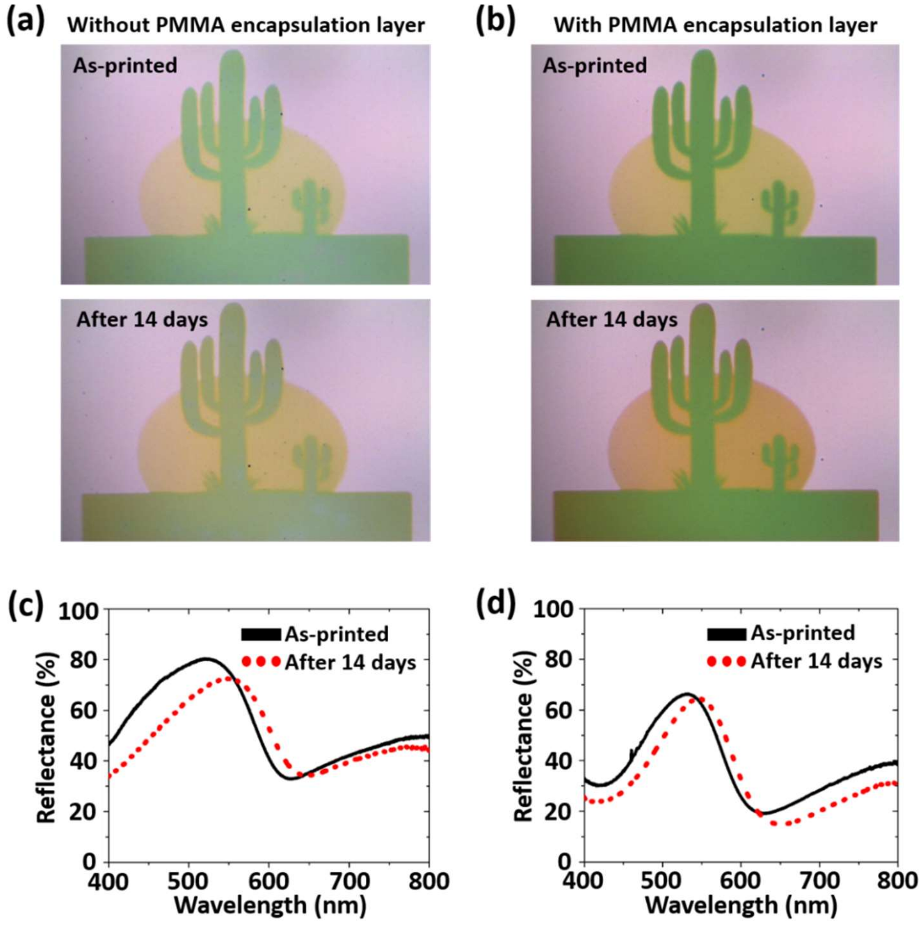

Figure S5. The effect of encapsulation layer on color retention time. The microscopic images of printed ‘Cactus’ (a) without PMMA encapsulation layer and (b) with PMMA encapsulation layer. The measured relative reflectance of (c) without PMMA encapsulation layer and (d) with PMMA encapsulation layer.

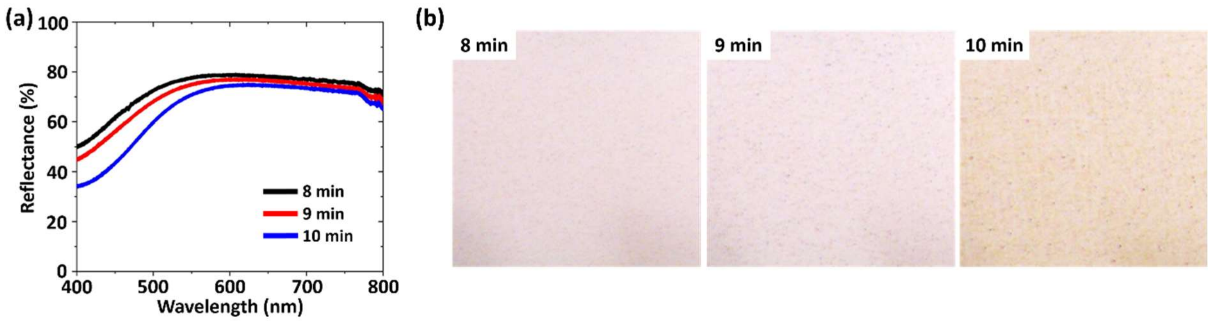

Figure S6. Characterization of the PPD reflector. (a) Measured relative reflectance of the thick PPD Ag films prepared on fused silica substrate with various printing time. (b) Optical images of the time-dependent printed PPD films.

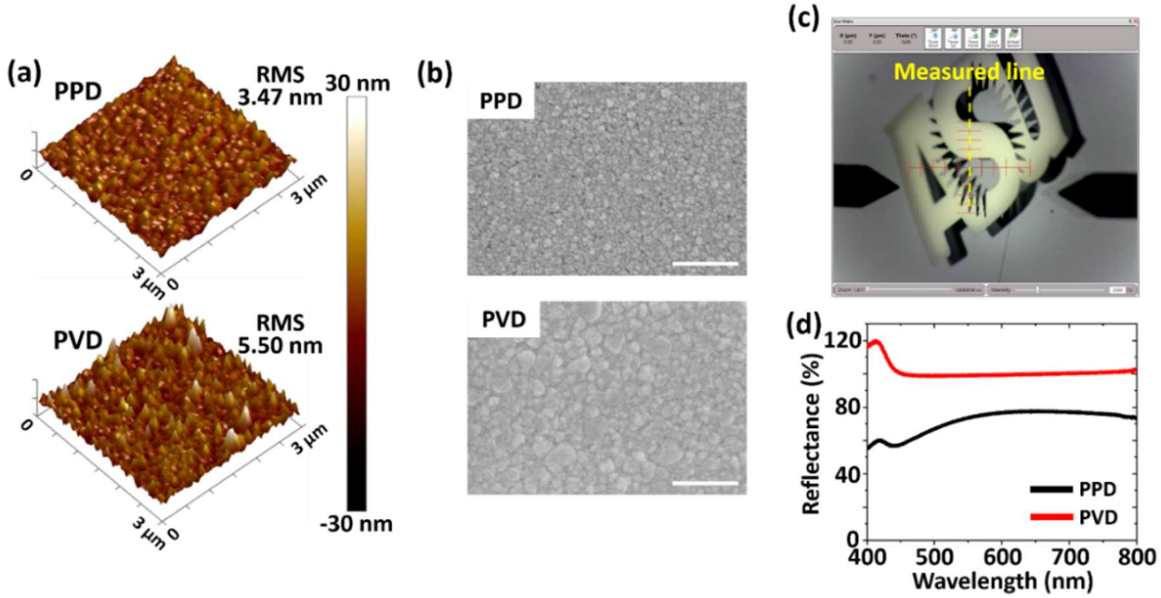

Figure S7. Characterization of the PPD back reflector. (a) 3D profile of AFM images and (b) SEM images (scale bar: 500 nm) of the 85 nm Ag films prepared on fused silica substrate by PPD and PVD, respectively. (c) Optical image of the 85 nm printed ASU logo under surface profiler. (d) Measured relative reflectance of the 85 nm Ag films prepared on fused silica substrate by PPD and PVD, respectively.

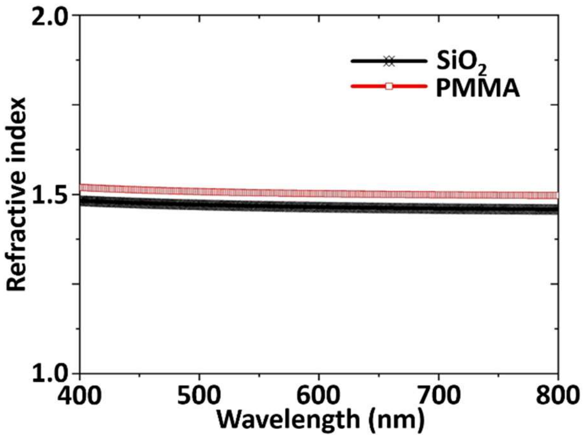

Figure S8. Measured refractive index of SiO_2 and PMMA by using UV-NIR spectroscopic ellipsometry.

Effective medium theory

In the classical model of free electron metals, the damping (γ) is determined by the scattering of the electrons with phonons, lattice defects, or impurities[2]. However, when particle size is comparable or smaller than the mean free path of the conduction electrons in the bulk material, scattering of the conduction electrons from the particle surface results in reduced effective mean free path (L_{eff}) and increased γ through the relation:

$$\gamma(L_{eff}) = \gamma_0 + \frac{Av_F}{L_{eff}} \qquad (1.1)$$

where γ_0 is the electron relaxation rate in the bulk material, v_F is the Fermi velocity and A is a dimensionless fitting parameter related to scattering[3]. Take account of this phenomenon, the equation to calculate the permittivity of finite-sized metal nanoparticles (ε_{np}) must be modified as

$$\varepsilon_{np}(\omega) = \varepsilon_{bulk}(\omega) + \frac{\omega_p^2}{\omega(\omega + i\gamma_0)} - \frac{\omega_p^2}{\omega(\omega + i\gamma)}. \qquad (1.2)$$

Here, ω is the frequency of incident light, ε_{bulk} is the permittivity for a bulk material, and ω_p is the plasma frequency[4]. In our case, we assume that AgNPs have spherical shape, hence the modified effective mean free path ($L_{eff} = 0.82R$, where R is the radius of nanoparticle) is used for the calculation[5]. Fig. S9 shows ε_{np} of spherical AgNPs with various radius. In the visible wavelength range, the particle size effects are more strongly manifested in the imaginary part, while the real part indicates a very minimal differences. Since our PPD film acts as the absorbing material in the FP cavity, it is important to have better understanding of the correlation between particle size and permittivity which is closely related to the absorbance of materials.

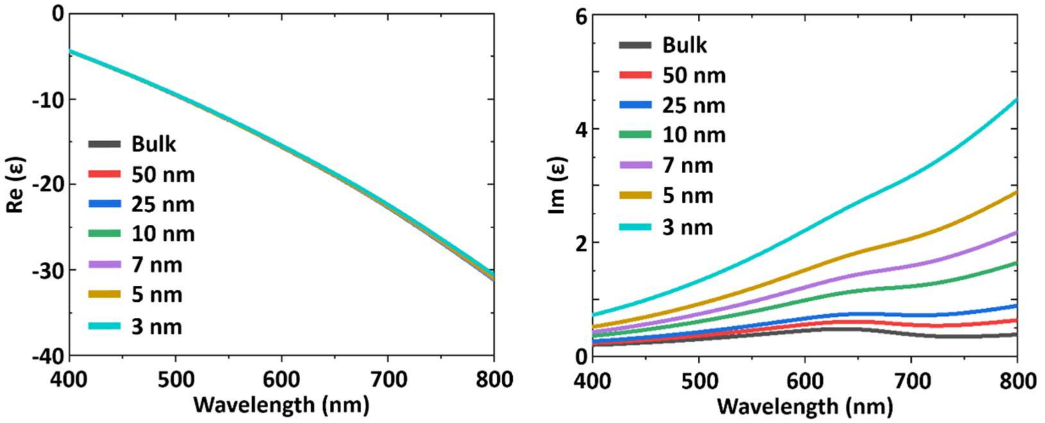

Figure S9. Size-dependent complex permittivities of spherical AgNPs as a function of wavelength. Left: Real part. Right: Imaginary part.

Effective medium theory (EMT) has been widely used to characterize optical properties of inhomogeneous materials, e.g. metal/polymer nanocomposites[6]. Maxwell-Garnett introduced a separated-grain structure whose inclusion material is dispersed in a continuous host material, while

Bruggeman suggested an aggregate structure which is filled with random mixture of the two constituents[7]. Since the reduction and polymer-assisted aggregation of AgNPs occur simultaneously in PPD process, we employ Bruggeman`s model to calculate the effective permittivity (ε_{eff}) of PPD film. The formula for ε_{eff} reads:

$$\varepsilon_{eff} = \varepsilon_p \left[1 + \frac{f(\varepsilon_{np} - \varepsilon_p)}{\varepsilon_p + n(1-f)(\varepsilon_{np} - \varepsilon_p)}\right] \qquad (1.3)$$

where ε_p = 1.91[8] is the permittivity of polymer (pAAm) and f is the filling factor of AgNPs in the nanocomposite[9]. The shape of AgNPs is very irregular in the real case (Fig. 2b), thus a shape factor n is introduced as a fitting parameter to generalize the equation. To investigate the average AgNP size, we performed pAAm concentration dependent PPD printing. From the SEM images with various pAAm concentration (Fig. S11), we observed particles from < 5 nm to 10 nm with distinguishable contrast, particularly in 30 mM of pAAm concentration, which probably attributed to higher pAAm capping efficiency for AgNPs (Fig. S11e). Therefore, the average size of AgNPs was set as 10 nm in diameter for the purpose of calculating ε_{np} using our model. The measured complex permittivity ($\varepsilon_m = \varepsilon_{re} + i\varepsilon_{im}$) of PPD film is obtained from the extracted spectroscopic ellipsometry data (Fig. 2d) through the relations, $\varepsilon_{re} = n^2 - k^2$ and $\varepsilon_{im} = 2nk$ for real and imaginary part, respectively. Fig. S10 shows ε_m and ε_{eff} of PPD film according to f, where n is set as 4.1. Importantly, when f is 0.81 (short-dotted pink line), calculated ε_{eff} is comparable to measured ε_m, which implies highly concentrated AgNPs in the PPD film. Consequently, those densely packed AgNPs absorb significant amount of incident light, which yields strong modulation of reflectance spectra in the visible wavelength range.

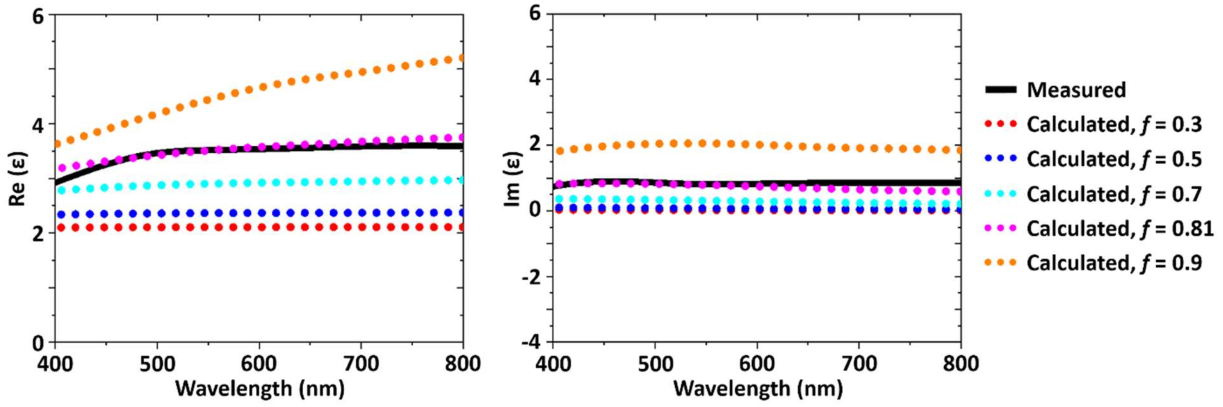

Figure S10. Measured complex permittivity (solid line) and calculated effective permittivity (dotted line) of AgNPs/pAAm nanocomposites as a function of wavelength. Calculation performed according to filling factor *f*. Left: Real part. Right: Imaginary part.

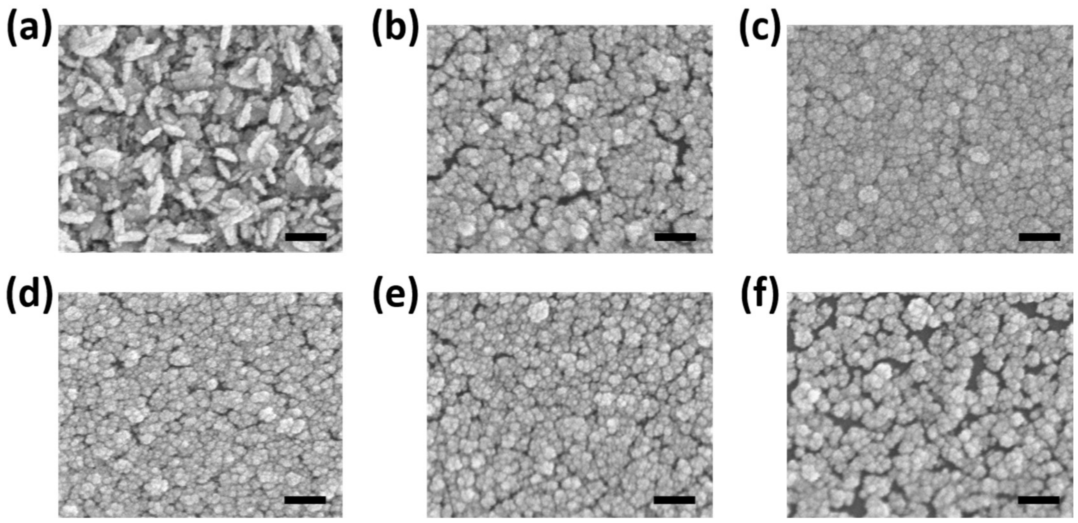

Figure S11. The effect of pAAm concentration on PPD film. (a) 0 mM, (b) 10 mM, (c) 16 mM, (d) 20 mM, (e) 30 mM, and (f) 40 mM of pAAm was mixed in Ag precursor solution, respectively, for printing PPD film (scale bar: 100 nm).

Reference

1 Singho, N. D., Johan, M. R., Lah, N. A. C., Temperature-dependent properties of silver-poly(methylmethacrylate) nanocomposites synthesized by in-situ technique. *Nanoscale Research Lett.* **9**:42 (2014).

2 Vollmer, M. & Kreibig, U. Optical Properties of Metal Clusters 1st edn, Vol. 25 (eds Toennies, J. P. et al.) Ch. 2 (Springer, 1995).

3 Coronado, E. A., Schatz, G. C., Surface plasmon broadening for arbitrary shape nanoparticles: A geometrical probability approach. *J. Chem. Phys.* **119**, 3926-3934 (2003).

4 Rashetnyak, V. Y. et al., Effective medium theory for anisotropic media with plasmonic core-shell nanoparticle inclusions. *Eur. Phys. J. Plus* **133**, 373 (2018).

5 Barma, M., Subrahmanyam, V., Optical absorption in small metal particles. *J. Phys.: Condens. Matter* **1**, 7681-7688 (1989).

6 Cai, W., Shalaev, V., Optical Metamaterials: Fundamentals and Applications 2010th edn, Ch. 2 (Springer, 2010).

7 Niklasson, G. A., Granqvist, C. G., Hunderi, O., Effective medium models for the optical properties of inhomogeneous materials. *Appl. Opt.*, **20**, 26-30 (1981).

8 Bardini, L. et al., Electrochemical Polymerization of Allylamine Copolymers. *Langmuir*, **29**, 3791-3796 (2013).

9 Rao, Y., Qu, J., Marinis, T., Wong, C. P. A Precise Numerical Prediction of Effective Dielectric Constant for Polymer-Ceramic Composite Based on Effective-Medium Theory. *IEEE Trans. Compon. Packaging Manuf. Technol.*, **23**, 680-683, (2000).
